# On the Connection Between Language Control and Executive Control—An ERP Study

**DOI:** 10.1162/nol_a_00032

**Published:** 2021-12-23

**Authors:** Mathieu Declerck, Gabriela Meade, Katherine J. Midgley, Phillip J. Holcomb, Ardi Roelofs, Karen Emmorey

**Affiliations:** School of Speech, Language, and Hearing Sciences, San Diego State University, San Diego, USA; Donders Institute for Brain, Cognition and Behaviour, Radboud University, Nijmegen, Netherlands; Department of Linguistics and Literary Studies, Vrije Universiteit Brussel, Brussels, Belgium; Joint Doctoral Program in Language and Communicative Disorders, San Diego State University & University of California, San Diego, USA; Department of Psychology, San Diego State University, San Diego, USA

**Keywords:** language control, executive control, language switching, task switching, ERPs

## Abstract

Models vary in the extent to which language control processes are domain general. Those that posit that language control is at least partially domain general insist on an overlap between language control and executive control at the goal level. To further probe whether or not language control is domain general, we conducted the first event-related potential (ERP) study that directly compares language-switch costs, as an index of language control, and task-switch costs, as an index of executive control. The language switching and task switching methodologies were identical, except that the former required switching between languages (English or Spanish) whereas the latter required switching between tasks (color naming or category naming). This design allowed us to directly compare control processes at the goal level (cue-locked ERPs) and at the task performance level (picture-locked ERPs). We found no significant differences in the switch-related cue-locked and picture-locked ERP patterns across the language and task switching paradigms. These results support models of domain-general language control.

## INTRODUCTION

A contested claim in the bilingual literature is that language control, which involves reducing cross-language interference and increasing the chances of selecting a word from the target language, is part of a more general executive control process (e.g., de Bruin et al., 2014; Green, 1998; Weissberger et al., 2015; however, see Calabria et al., 2015; Declerck et al., 2015; Jylkkä et al., 2018). The absence of conclusive evidence for domain-general language control could be due to prior research typically not focusing on the processing level at which language control and executive control are assumed to overlap, namely the goal level (e.g., Green, 1998; Roelofs, 2003; Thomas & Allport, 2000). In the present study, we investigated the claim that language control is domain general by using event-related potentials (ERPs) to examine whether or not there is overlap between language control and executive control during goal activation and selection, and/or during later stimulus processing.

The literature on whether or not language control is domain general is quite divided. For instance, Allport and colleagues (Meuter & Allport, 1999; Thomas & Allport, 2000) proposed that language control occurs entirely between language task schemas. Task schemas are domain-general mental processes used to achieve a specific goal (e.g., produce in a specific language or perform a specific task). Put differently, these authors assumed that language control occurs entirely at the goal level, and that it is part of executive control. Others have proposed that this task schema mechanism is complemented by a language-specific control process that occurs at the lemma level between translation-equivalent representations (e.g., Green, 1998). Still others have assumed that language control occurs entirely at the lemma level rather than at the goal level (Declerck et al., 2015; Grainger et al., 2010). More specifically, Declerck et al. (2015) proposed that language control occurs between language nodes, which are the mental representations of each language at the lemma level, and translation-equivalent representations at the lemma level. Thus, the mechanisms that underlie language control and the extent to which they are domain general versus language specific continue to be debated.

To examine the possibility of domain-general language control, most studies have compared performance in language switching, to examine language control processes, and task switching, to examine executive control processes (for other methodologies used to examine domain-general language control see, e.g., Declerck et al., 2019; Jylkkä et al., 2018; Linck et al., 2020; Struys et al., 2019). In a language switching paradigm, bilinguals typically name digits or pictures based on a visually presented language cue (e.g., frames in a different color for each language). This setup requires bilinguals to either switch from one language to another across trials (switch trials) or repeat the same language (repetition trials). The cost in response time and/or accuracy of switching languages—relative to staying in the same language—has been used as an index of language control (e.g., Declerck & Philipp, 2015; Green, 1998). Task-switch costs are a measure of executive control that are obtained using a similar setup as the language-switch costs (e.g., Kiesel et al., 2010). However, instead of switching between different languages, participants switch between distinct tasks (e.g., switching between parity and magnitude judgments with number stimuli).

Even though some studies comparing language- and task-switch costs have shown a similar pattern across the two paradigms (e.g., Declerck et al., 2017; Weissberger et al., 2015), others have not (e.g., Branzi et al., 2016; Calabria et al., 2015). It could be that language control is only partly domain general, as assumed by the inhibitory control model (Green, 1998). If so, some studies may have failed to provide evidence for domain-general language control because they did not sufficiently capture the main locus of the overlap between language and executive control. As discussed, this overlap is assumed to occur at the goal level (e.g., Green, 1998; Roelofs, 2003; Thomas & Allport, 2000). Several studies allow for insight into whether there is an overlap between language and executive control at the goal level by examining the cue-to-stimulus interval in language and task switching. During this interval, participants activate and, given sufficient time, select the goal to speak a specific language during language switching or the goal to perform a specific task during task switching. This makes the cue-to-stimulus interval particularly interesting to examine possible links between language and executive control.

Two ERP language-switching studies that examined cue processing are relevant here. Lavric et al. (2019) asked German-English bilinguals to perform a picture naming task in mixed language blocks. The cue-to-stimulus interval was either 100 or 1,500 ms. When focusing on the cue-locked ERPs with a cue-to-stimulus interval of 1,500 ms, they observed a posterior switch positivity (time window: 300–700 ms). That is, cues in switch trials elicited a larger positivity across posterior electrodes than cues in repetition trials. The posterior switch positivity is interpreted as an index of task rule activation and is often observed in the task switching literature (e.g., Barceló & Cooper, 2018; Chevalier et al., 2015; Manzi et al., 2011; for reviews, see De Baene & Brass, 2014; Karayanidis et al., 2010). This finding led Lavric et al. (2019) to conclude that there is an overlap between control processes implemented on task schemas, and thus at the goal level, during bilingual language production and non-linguistic processing.

However, this finding of Lavric et al. (2019) was not supported by the ERP language-switching study of Verhoef et al. (2010). With a cue-to-stimulus interval of 750 ms, Verhoef and colleagues found an anterior switch negativity around the same time window as Lavric et al. observed a posterior switch positivity. Cues in switch trials elicited a larger negativity across anterior electrodes than cues in repetition trials. Lavric et al. also observed an anterior negativity that occurred after their posterior positivity and was relatively more protracted in time. These differences in timing led to different accounts of the anterior switch negativity: Verhoef et al. proposed that the earlier anterior switch negativity was an index of goal engagement, whereas Lavric et al. assumed that it was an index of goal maintenance.

Yet the findings of Lavric et al. (2019) and Verhoef et al. (2010) might not be as different as they initially appear. In an earlier task switching study, Lavric et al. (2008) found a dipolar ERP component related to cue processing, with an anterior switch negativity and a posterior switch positivity that occurred in parallel and were interdependent. Hence, the posterior switch positivity observed by Lavric et al. (2019) and the anterior switch negativity observed by Verhoef et al. (2010) might actually be two parts of the same dipolar ERP component.

Verhoef et al. (2010) also observed a negative peak in an earlier time window (200–350 ms) with cue-locked ERPs that was larger for switch than repetition trials over posterior sites. According to the authors, this component is an index of disengagement of the non-target language. In the cue-locked task switching literature, there is typically an early posterior switch positivity, which can occur as early as 200 ms but shows large temporal variability across studies (e.g., Eppinger et al., 2007; Jamadar et al., 2010; Manzi et al., 2011). This early posterior switch positivity has been linked to task goal activation, and can be dissociated from the later posterior switch positivity (e.g., Eppinger et al., 2007; Manzi et al., 2011; for reviews, see De Baene & Brass, 2014; Karayanidis et al., 2010).

We have mainly focused thus far on cue processing since the cue-locked ERPs provide theoretically interesting insights into domain-general language control. However, the literature indicates that there might also be some overlapping processes across language and executive control that occur after the target stimulus has been presented. Most prominently, a similar stimulus-locked N2 component has been observed in both language and task switching studies, which has generally been taken as an index of inhibitory control of the non-target language/task and conflict monitoring (e.g., Gaál & Czigler, 2015; Finke et al., 2012; Jackson et al., 2001; Kang et al., 2020). This component entails a larger negativity for switch trials than repetition trials and is usually found around 200–350 ms after stimulus presentation. Unlike the cue-locked, early posterior negativity observed in Verhoef et al. (2010), the stimulus-locked N2 is typically more pronounced over anterior sites (however, see Zheng et al., 2020).

Stimulus-locked ERPs in the language switching paradigm are less straightforward beyond the N2 component. Some studies have observed a late positive complex (LPC), characterized by a larger positivity over posterior sites for switch trials relative to repetition trials around 400–650 ms after stimulus presentation (e.g., Jackson et al., 2001; Liu et al., 2016; Martin et al., 2013), whereas others have found a widespread late switch-related negativity (Kang et al., 2020; Peeters, 2020; Peeters & Dijkstra, 2018). Task switching studies, on the other hand, tend to find evidence for a P3b component around 400–600 ms after stimulus onset (e.g., Barceló, 2003; Provost et al., 2018; Tieges et al., 2007; for a review, see Gajewski et al., 2018). This component is generally characterized by a larger positivity for repetition trials than switch trials over posterior sites. In sum, across the language and task switching literatures, there are three different post-N2 stimulus-locked ERP patterns that temporally overlap and that might be shared by language and executive control: the LPC, a late switch-related negativity, and the P3b component.

As this brief review suggests, it is not yet clear whether similar control processes are implemented during cue processing or subsequently during stimulus processing between language and task switching. It is especially difficult to draw conclusions across studies from the language and task switching literatures due to the different experimental designs and approaches to analyzing and reporting results. Additionally, the relatively small number of language-switching ERP studies, especially with regard to cue processing, makes it difficult to gauge the robustness of their results. Hence, to further investigate the possibility of domain-general language control with ERPs, it would be beneficial to directly compare language and task switching patterns within the same participants and using the same experimental design (e.g., cue-to-stimulus interval, stimuli, responses). In the current study, we did exactly that, by conducting the first ERP study that directly compares control processes in nearly identical language and task switching paradigms. More specifically, in the language-switching blocks, English-Spanish bilingual participants named either the color (block X) or category (block Y) of pictures in a mixed language context (English vs. Spanish). In the task-switching blocks, the same bilinguals used either English (block X) or Spanish (block Y) in a mixed task context (color vs. category naming). So the only difference between the language and task switching paradigms was that bilingual participants switched between languages in the former and between tasks in the latter (cf. Declerck et al., 2017). By having such similar language and task switching paradigms, we were able to directly compare the temporal neural dynamics during language and task switching for the first time.

Since the language and task switching paradigms are methodologically identical, differences in the ERP pattern between language- and task-switch costs could be taken as evidence of different underlying mechanisms for language control and executive control. For instance, if different control processes are implemented during goal processing, then differences in the ERP pattern should be observed during cue processing. According to the limited literature, a larger early negativity in the cue-locked ERPs might be expected during language switching (Verhoef et al., 2010), whereas a larger early posterior positivity might be expected during task switching (e.g., Eppinger et al., 2007; Jamadar et al., 2010; Manzi et al., 2011). Moreover, in the later time window of the cue-locked ERPs, it could be that switching languages results in a larger anterior negativity (Verhoef et al., 2010), whereas switching tasks results in a larger posterior positivity (for reviews, see De Baene & Brass, 2014; Karayanidis et al., 2010) or a dipolar component (Lavric et al., 2008). Finally, if different control mechanisms are involved for language and task switching we might also expect a difference in a late time window of the picture-locked ERPs. Task switching studies tend to observe a modulation of the P3b component (for a review, see Gajewski et al., 2018), whereas the language switching literature has shown a switch-related modulation of the LPC (e.g., Jackson et al., 2001; Liu et al., 2016; Martin et al., 2013) or a late switch-related negativity (Kang et al., 2020; Peeters, 2020; Peeters & Dijkstra, 2018).

## METHOD

### Participants

Twenty-six English-Spanish bilinguals took part in the experiment. Two participants were excluded due to experimenter error. The remaining 24 participants consisted of 22 women who were 23.2 years old on average (*SD* = 3.0 years). All participants were right-handed, no older than 30 years of age, and no one had a prior history of neurological dysfunctions. Prior to the experiment the participants were asked to complete a language history questionnaire and an English and Spanish vocabulary test through a lexical decision task (LexTALE; Izura et al., 2014; Lemhöfer & Broersma, 2012; see Table 1). All participants were volunteers who were paid for their time. Informed consent was obtained in accordance with the Institutional Review Board at San Diego State University.

**Table 1.** Means for the demographic information (*SD* in parentheses) for each language

	English	Spanish
Age of acquisition (years)	4.6 (3.7)	1.9 (3.8)
Time currently used (%)	63.2 (28.3)	36.8 (28.3)
Speaking*	6.6 (0.9)	5.9 (1.2)
Writing*	6.5 (1.0)	5.5 (1.3)
Reading*	6.6 (0.8)	5.9 (1.2)
LexTALE (%)	88.7 (9.2)	74.8 (14.0)

*Note*. * Self-rated scores on a scale of 1 (low proficiency) to 7 (high proficiency).

### Stimuli

The stimuli consisted of 48 pictures (line drawings) that were presented in one of four colors (brown, green, blue, and orange), with an equal number of pictures in each color. Each of the pictures depicted a concept from one of four semantic categories (furniture, clothing, food, and animals), with 12 pictures designated to each category.

The participants were instructed to use one of two languages (English or Spanish) or perform one of two tasks (color naming or category naming) based on one of four shape cues (square, circle, pentagon, and parallelogram). The cues subtended a maximal visual angle of 2.7° in each direction. The square and circle cues were always used together in the same block, as were the pentagon and parallelogram, because these shapes were easily distinguishable from one another. Furthermore, one set of cues was always used in the language switching paradigm, and the other set in the task switching paradigm for a specific participant. The mapping between cues and languages/tasks was counterbalanced across participants.

### Procedure

**Figure 1.**
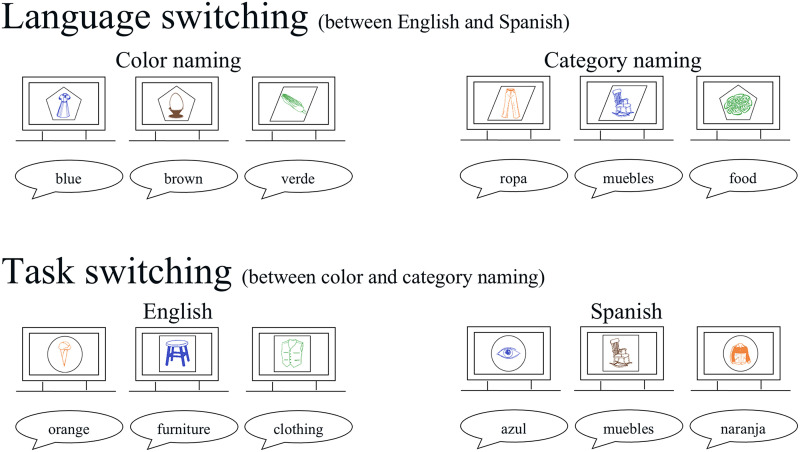
Overview of the different experimental conditions and blocks. In this example, the pentagon was the cue for participants to respond in English and the parallelogram was the cue to respond in Spanish in the language switching paradigm. The circle was the cue to name the color in which the picture was presented, and the square was the cue to name the semantic category of the picture in the task switching paradigm. It should also be noted that the size of the cues and pictures relative to the screen are much larger in this figure than in the actual experiment, to make them more visible.

The experiment took place in a dimly lit room, where the participants were seated in a comfortable chair about 150 cm from the monitor. Prior to each of the four experimental blocks, each consisting of 96 trials, there was a practice block of 20 trials. In the two language-switching blocks, the cue determined whether participants should use English or Spanish. In one of the blocks, the bilingual participants named the color in which the picture was presented and in the other block they named the semantic category of the picture (See Figure 1 for an overview of the different blocks). The order of these two blocks was counterbalanced across participants. In the task-switching blocks, the cue determined whether participants should name the color in which the picture was presented or its semantic category. One block was entirely in English and the other in Spanish. The order of these two blocks was also counterbalanced across participants. Finally, the order of the switching paradigm that came first (i.e., language or task) was also counterbalanced across participants.

**Figure 2.**
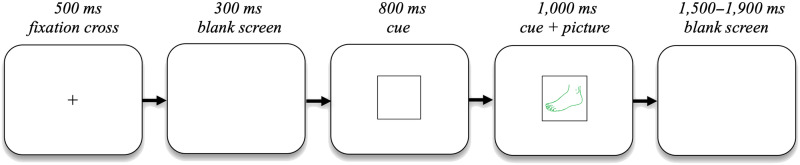
Overview of the trial procedure.

Each trial started with a central fixation cross that was presented for 500 ms. This was followed by a blank screen for 300 ms, after which the cue shape was presented. After 800 ms of solely the cue shape being on the screen, the picture was presented inside the cue shape for 1,000 ms. Finally, a blank screen appeared for 1,500 ms with a jitter of 0–400 ms (see Figure 2 for a visual depiction of the trial procedure). The participants were asked to blink after they responded up until the fixation cross.

### Behavioral Analyses

The independent variables of the behavioral analyses were Paradigm (language vs. task switching) and Trial type (switch vs. repetition trials). The dependent variables for the behavioral analyses were reaction times and error rates. (The data have been made publicly available at https://osf.io/67mep/.)

For the error analysis, we only counted errors that were not preceded by other errors or omissions; otherwise, it would be unclear whether to consider the error trial as a switch or repetition. The errors consisted of incorrect use of language or incorrect target word, and were noted by a trained research assistant who was fluent in both languages. Reaction times were calculated from picture presentation to voice onset and were only included in the analysis if they were between 200 and 2,500 ms long. The first trial of each block and error trials were excluded from the RT analysis, as were trials following an error trial or an omission trial for the reason discussed above. These criteria resulted in the exclusion of 11.01% of trials.

### EEG Recording and Analyses

Participants were fitted with Electro-Caps using 29 active electrodes. Additional electrodes were placed on each mastoid, under the left eye, and next to the outer canthus of the right eye. The recording from the left mastoid was used as a reference, both during recording and for all analyses. The recording from the right mastoid was used to measure differential mastoid activity (there were no differences between conditions at the right mastoid site, so the left mastoid was used as a reference for all subsequent ERP comparisons). The electrode below the left eye was used to identify blinks, in combination with the activity of FP1, and the electrode next to the right eye was used to identify any horizontal eye movements. Impedances were maintained below 2.5 kΩ. EEG was amplified using SynAmps RT amplifiers (Neuroscan-Compumedics) with a bandpass of DC to 100 Hz and was sampled throughout at 500 Hz.

For the cue-locked ERP data, each epoch was time-locked to cue onset and was 900 ms long, including 100 ms prior to the cue as a baseline. For the picture-locked ERP data, each epoch was time-locked to stimulus onset and was 700 ms long, including 100 ms prior to the stimulus as a baseline. The length of the picture-locked epoch was determined based on the shortest reaction times to minimize speech-related artifacts. Any artifacts, including blinks or other eye movements, that were detected resulted in exclusion of that specific trial from the corresponding analyses. Additionally, the first trial of each block, error trials, and trials immediately following these errors were excluded from analyses. These criteria resulted in the exclusion of an average of 18.45% of trials from the cue-locked analyses and 20.19% of trials from the picture-locked analyses. More specifically, there was an average of 75.79 (*SD* = 12.34) language-switch trials, 80.33 (*SD* = 10.80) language-repetition trials, 78.75 (*SD* = 9.00) task-switch trials, and 78.29 (*SD* = 12.21) task-repetition trials per participant in the cue-locked ERP analyses. In the picture-locked ERP analyses, there was an average of 74.54 (*SD* = 10.99) language-switch trials, 77.79 (*SD* = 9.09) language-repetition trials, 76.79 (*SD* = 8.63) task-switch trials, and 77.33 (*SD* = 9.85) task-repetition trials per participant.

**Figure 3.**
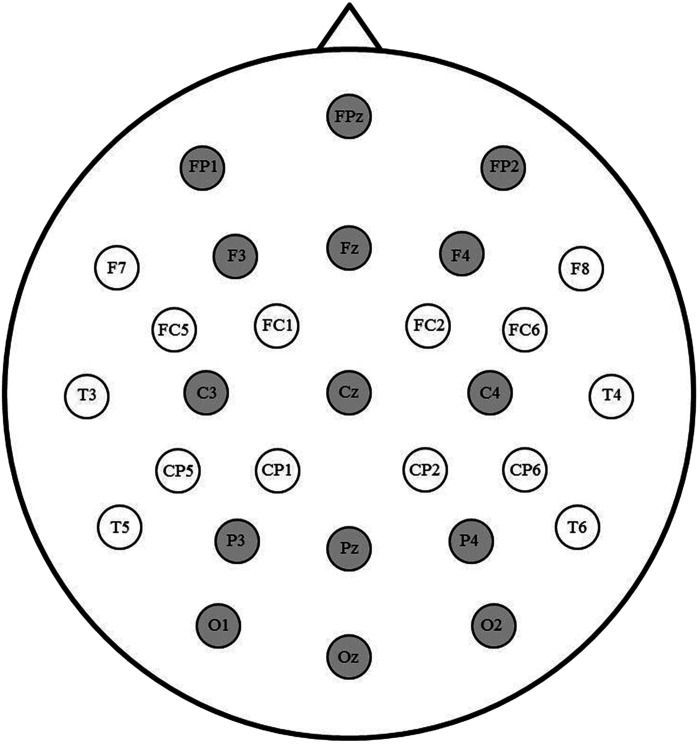
Sites highlighted in grey were included in the analyses.

Separate ERPs were averaged for each participant and condition at each electrode and low-pass filtered at 15 Hz. Mean amplitudes of the early time window in the cue-locked data were calculated for each participant between 200 and 350 ms (cf. Verhoef et al., 2010). We were also interested to see whether a posterior switch positivity, an anterior switch negativity, or a dipolar component would be observed in a later time window in the cue-locked data, which we measured between 350–700 ms (cf. Lavric et al., 2019). Regarding the picture-locked time windows, we also calculated the mean amplitudes of the early time window between 200 and 350 ms, in line with the N2 literature (e.g., Gaál & Czigler, 2015; Zheng et al., 2020). A late picture-locked time window was calculated between 400–600 ms, which overlaps with the time windows of the LPC, late switch-related negativity, and the P3b (e.g., Gajewski et al., 2018; Jackson et al., 2001; Kang et al., 2020). To capture patterns at both anterior and posterior sites, we relied on a broad grid of 15 electrodes for the analyses, as illustrated in Figure 3. The omnibus ANOVA consisted of the following factors: Paradigm (language vs. task switching), Trial type (switch vs. repetition trials), Laterality (left, midline, right), and Anterior/Posterior (prefrontal, frontal, central, parietal, occipital). For all measures containing more than one degree of freedom in the numerator, we applied Greenhouse-Geisser correction.

## RESULTS

### Behavioral Results

The reaction time analysis showed a significant main effect of Trial type, with slower responses in switch trials (1,062 ms) than in repetition trials (1,019 ms), *F*(1, 23) = 33.17, *p* < 0.001, η_p_^2^ = 0.590 (see Table 2).

**Table 2.** Overall reaction times in ms and percentage of errors (*SD* in parentheses), as a function of Paradigm (language vs. task switching) and Trial type (switch vs. repetition trials)

Dependent variables	Language switching			Task switching		
	Switch	Repetition	Switch costs	Switch	Repetition	Switch costs
Reaction time	1,068 (203)	1,024 (184)	44	1,056 (201)	1,014 (206)	42
Error rate	5.22 (3.76)	2.11 (2.28)	3.11	3.62 (2.28)	2.70 (2.15)	0.92

No significant difference was observed between the size of language- and task-switch costs (95% CI = [−33, 36]; for further evidence along these lines obtained with an ex-Gaussian analysis of reaction time distributions, see Supplementary Materials; supporting information can be found online at https://www.mitpressjournals.org/doi/suppl/10.1162/nol_a_00032; the interaction between Paradigm and Trial type was not significantly influenced by the order of the paradigms, which was also the case for all other analyses.) To quantify this null effect, we relied on Bayesian Null Hypothesis Testing (e.g., Aczel et al., 2018; Rouder et al., 2009; Wagenmakers et al., 2018), which is a statistical test that shows the degree to which the Null hypothesis (H0) should be accepted over the Alternative hypothesis (H1). To this end, we compared a model that only includes main effects of Paradigm and Trial type against a model that includes both the main effects and their interaction. The results confirmed that a model that only includes both main effects accounts for the data better than a model that also includes the interaction (BF_01_ = 3.58; Kass & Raftery, 1995). In other words, we have statistical evidence that the language- and task-switch costs are about three and a half times more likely to be similar than they are to be different.

The error rate analysis showed a significant main effect of Trial type, with more errors observed in switch trials (4.39%) than in repetition trials (2.41%), *F*(1, 23) = 28.49, *p* < 0.001, η_p_^2^ = 0.553 (see Table 2). Furthermore, the interaction between Paradigm and Trial type was significant, *F*(1, 23) = 13.98, *p* = 0.001, η_p_^2^ = 0.378, with larger language-switch costs (3.11%) than task-switch costs (0.92%; 95% CI = [1.18, 3.56]).

### Cue-Locked ERP Results

**Figure 4.**
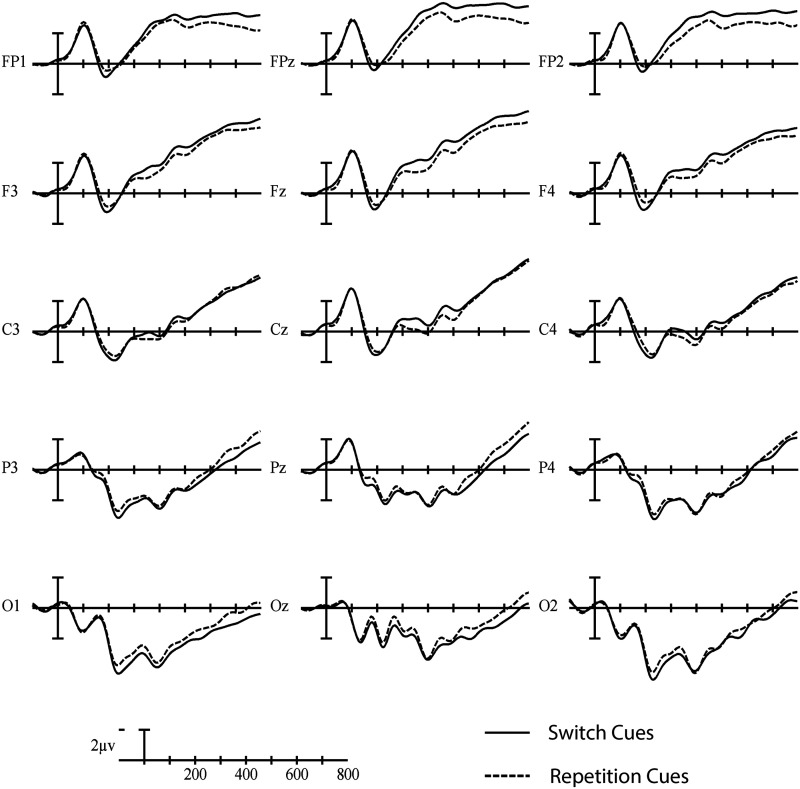
Grand average cue-locked ERP waveforms elicited across language and task switching with switch trials (solid line) and repetition trials (dashed line). Each vertical tick marks 100 ms, and negative is plotted up. The calibration bar marks 2 μV.

**Figure 5.**
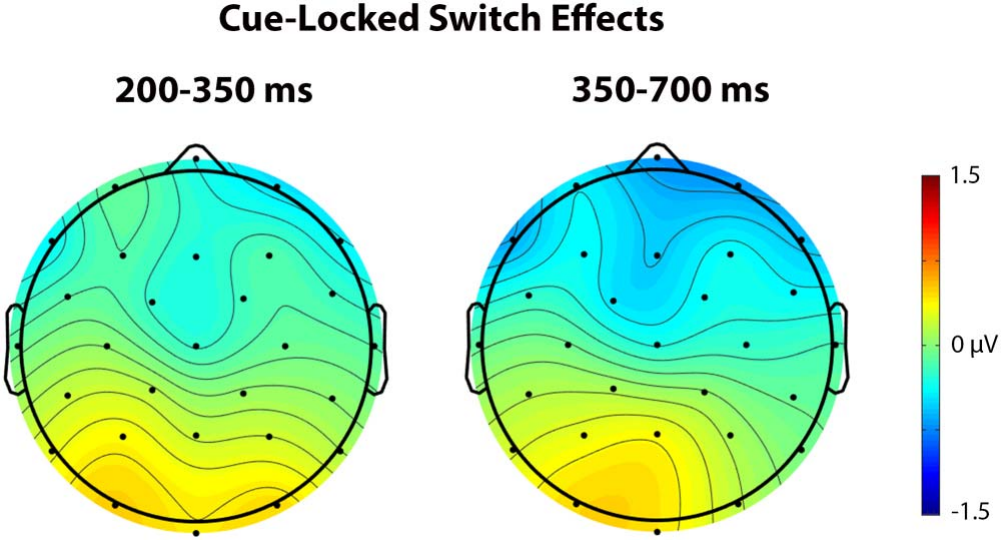
These scalp voltage maps show the distribution of the cue-locked Trial type effect (switch trials − repetition trials) in the 200–350 ms (left) and 350–700 ms (right) time windows collapsed across language and task switching paradigms. Cool colors indicate a larger negativity for switch trials relative to repetition trials.

In the early time window (200–350 ms), a significant interaction was observed between Trial type and Anterior/Posterior, *F*(4, 92) = 4.76, *p* = 0.018, η_p_^2^ = 0.172, indicating a larger negativity during cue processing in switch trials than repetition trials at anterior sites and a larger positivity in switch than repetition trials at more posterior sites (see Figures 4 and 5).

**Figure 6.**
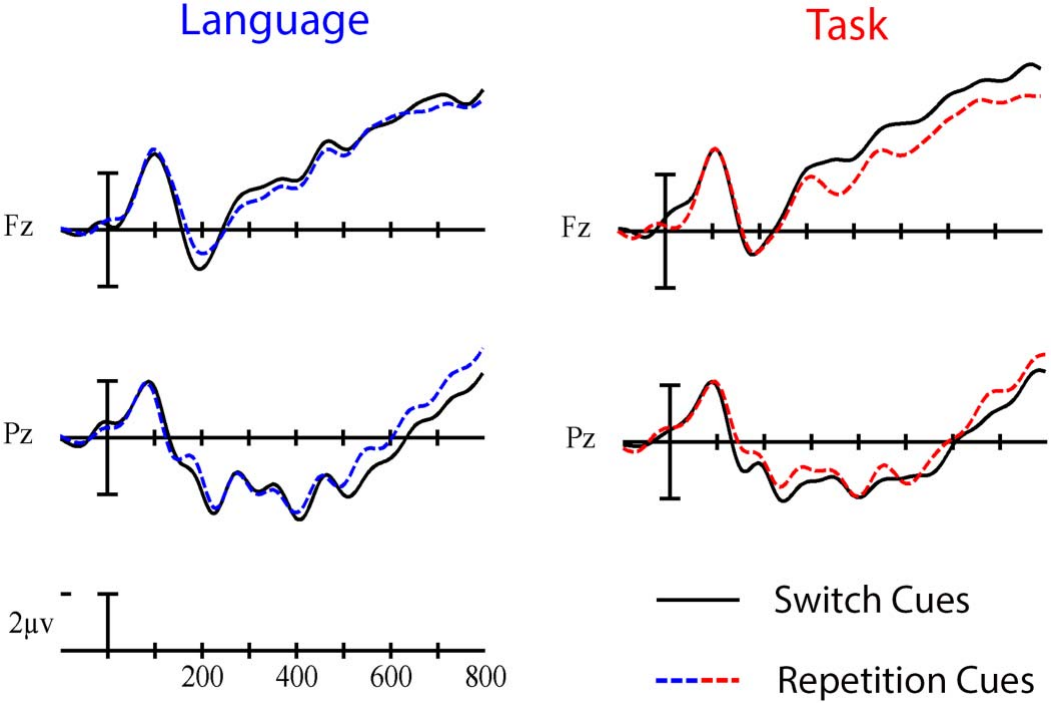
Grand average cue-locked ERP waveforms at representative electrodes Fz and Pz elicited by switch trials (solid line) and repetition trials (dashed line) in the language (left) and task switching (right) paradigms. Each vertical tick marks 100 ms, and negative is plotted up. The calibration bar marks 2 μV.

No significant switch cost differences were observed between language and task switching (95% CI = [−3.42, 1.08]). Using Bayesian Null Hypothesis Testing, we confirmed that a model that does not include the interaction between Paradigm and Trial type, but does include both main effects, accounts for the data better than a model that includes the interaction. This held true for both Fz (BF_01_ = 3.38; 95% CI = [−0.83, 4.81]) and Pz (BF_01_ = 3.18; 95% CI = [−5.73, 0.35]), which were chosen as representative anterior and posterior electrodes where the Trial type effects appeared largest across all cue- and picture-locked analyses (see the waveforms and voltage maps in this article). That is, we have statistical evidence that the language- and task-switch costs are about three times more likely to be similar than they are to be different in this time window (for a visual depiction, see Figure 6).

In the late time window (350–700 ms), a significant main effect was found for Paradigm, *F*(1, 23) = 6.01, *p* = 0.022, η_p_^2^ = 0.207, indicating that cues elicited a larger negativity in the task switching paradigm than in the language switching paradigm. There was also an interaction between Trial type and Anterior/Posterior, *F*(4, 92) = 4.59, *p* = 0.029, η_p_^2^ = 0.167, indicating that cues in switch trials elicited larger negativities than those in repetition trials over anterior sites, but larger positivities over posterior sites.

Again, no significant switch cost differences were found between language and task switching (95% CI = [−0.31, 1.08]). Using Bayesian Null Hypothesis Testing, we confirmed that a model that does not include the interaction between Trial type and Paradigm, but does include both main effects, accounts for the data better than a model that includes the interaction and both main effects for Pz (BF_01_ = 3.65; 95% CI = [−0.30, 1.73]). However, no definitive conclusions could be made for Fz (BF_01_ = 1.95; 95% CI = [−0.65, 0.99]) based on the Bayesian Null Hypothesis Testing (cf. Kass & Raftery, 1995). Hence, it is difficult to indicate one way or another whether there was a substantial difference in this window with respect to Trial type between language and task switching over anterior sites.

### Picture-Locked ERP Results

**Figure 7.**
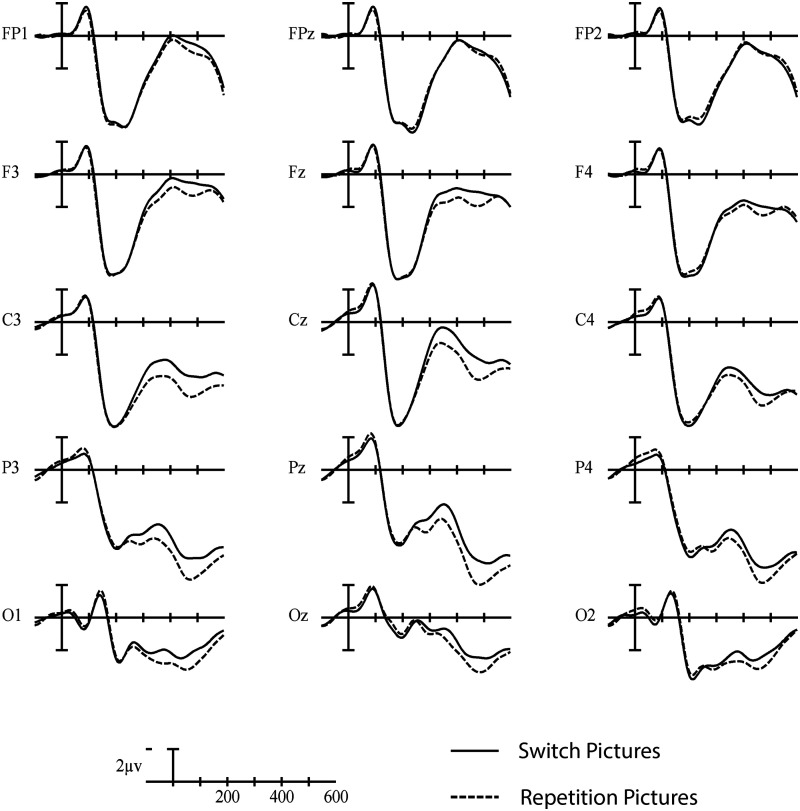
Grand average picture-locked ERP waveforms elicited across language and task switching with switch trials (solid line) and repetition trials (dashed line). Each vertical tick marks 100 ms and negative is plotted up. The calibration bar marks 2 μV.

**Figure 8.**
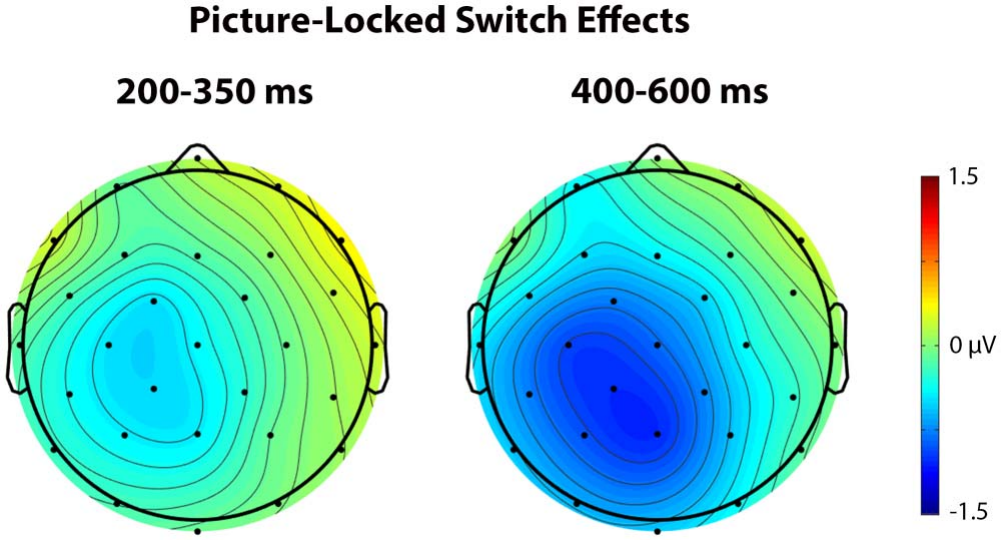
These scalp voltage maps show the distribution of the picture-locked Trial type effect (switch trials – repetition trials) in the 200–350 ms (left) and 400–600 ms (right) time windows collapsed across language and task switching paradigms. Cool colors indicate a larger negativity for switch trials relative to repetition trials.

In the early time window (200–350 ms), a significant interaction was observed between Trial type and Laterality, *F*(2, 46) = 6.57, *p* = 0.009, η_p_^2^ = 0.222, indicating a larger negativity in switch compared to repetition trials, especially over the left hemisphere (see Figures 7 and 8).

**Figure 9.**
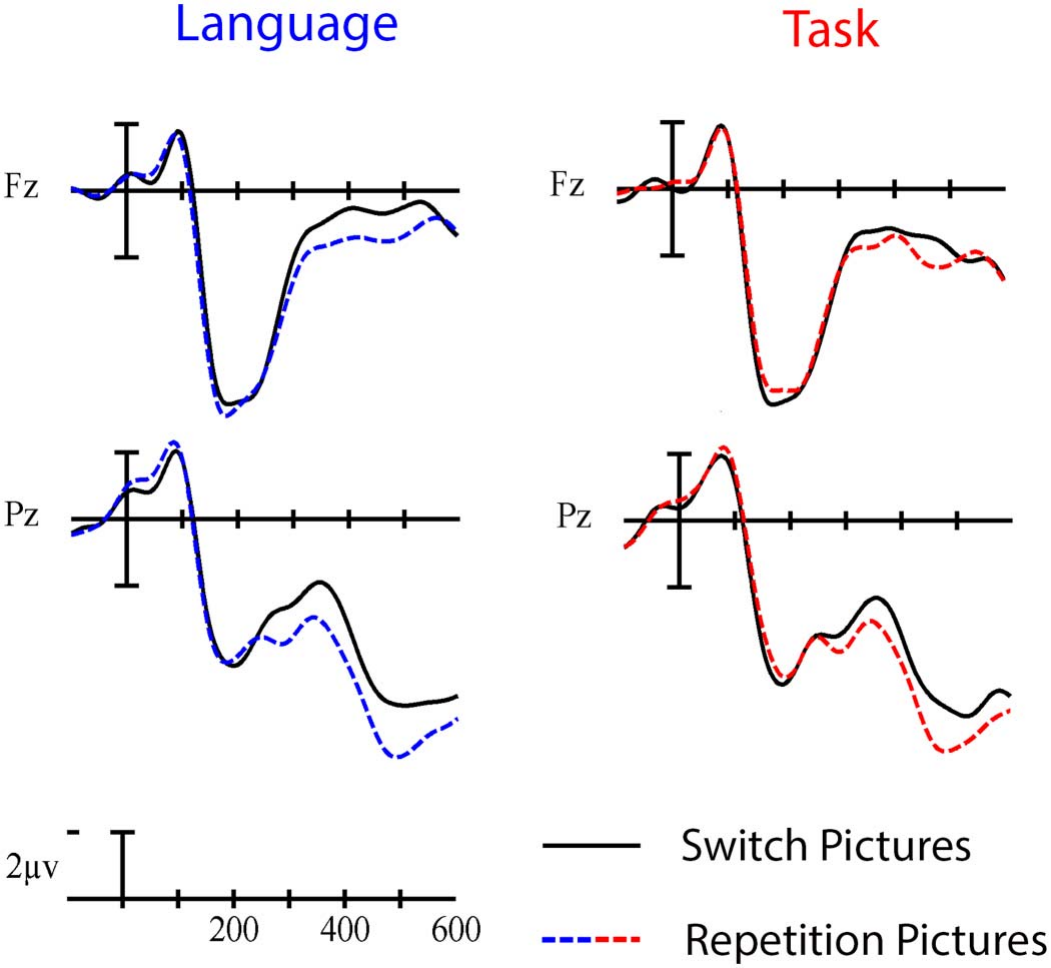
Grand average picture-locked ERP waveforms at representative electrodes Fz and Pz elicited by switch trials (solid line) and repetition trials (dashed line) in the language (left) and task switching (right) paradigms. Each vertical tick marks 100 ms, and negative is plotted up. The calibration bar marks 2 μV.

Similar to the cue-locked ERPs, no significant switch cost differences were observed between language and task switching (95% CI = [−1.01, 0.37]). Using Bayesian Null Hypothesis Testing, we confirmed that a model that does not include the interaction between Paradigm and Trial type, but does include both main effects, accounts for the data better than a model that includes the interaction at both Fz (BF_01_ = 3.19; 95% CI = [−1.31, 0.57]) and Pz (BF_01_ = 3.24; 95% CI = [−1.17, 0.60]) (for a visual depiction, see Figure 9).

In the late time window (400–600 ms), a significant interaction was observed between Paradigm and Anterior/Posterior, *F*(4, 92) = 5.91, *p* = 0.009, η_p_^2^ = 0.204, indicating a larger negativity when performing in the task switching paradigm compared to the language switching paradigm, especially over anterior sites. There was also a significant interaction between Trial type and Laterality, *F*(2, 46) = 4.99, *p* = 0.026, η_p_^2^ = 0.178, indicating a larger negativity in switch trials compared to repetition trials, especially over left hemisphere sites.

This analysis also showed no significant switch cost differences between language and task switching (95% CI = [−1.28, 0.71]). Using Bayesian Null Hypothesis Testing, we confirmed that a model that does not include the interaction between Paradigm and Trial type, but does include both main effects, accounts for the data better than a model that includes the interaction and both main effects at both Fz (BF_01_ = 3.32; 95% CI = [−1.68, 1.01]) and Pz (BF_01_ = 3.37; 95% CI = [−1.41, 0.96]).

## DISCUSSION

In the current ERP study, we examined whether or not the neurocognitive mechanisms underlying language control (for possible neural loci, see Kim et al., 2012; Sulpizio et al., 2020) are domain general by comparing switch costs between carefully matched language and task switching paradigms. We observed similar costs in reaction times for language and task switches, but there was a difference in error rate. Most importantly, we obtained similar cue-locked and picture-locked ERP patterns across the language and task switching paradigms. In what follows, we first discuss the behavioral results, followed by the cue-locked and picture-locked results. Finally, we discuss the limitations of this study.

### Behavioral Data

We did not find any significant switch cost differences in reaction time between the language switching and task switching paradigms (see also Supplementary Materials). This result was further supported by Bayesian Null Hypothesis Testing, which provided statistical evidence to support the conclusion that language- and task-switch costs were similar. Such a finding is in line with the hypothesis that language control is domain general. The error rate data were more difficult to interpret. Although the switch costs were larger during language switching relative to task switching, this result must be qualified by the fact that the error rates were quite low overall (3.3% of all trials).

If reliable, the error rate pattern could be interpreted as evidence for differences in the processes that are engaged in language versus executive control. Another possibility is that the quantitative difference that we observed could reflect a difference in the extent to which the same control process is engaged across paradigms. For instance, language switching might require more control processes than task switching (e.g., more inhibition of the non-target language than of the non-target task), which could have resulted in larger language-switch costs. This is plausible since the mental representations of the first and second languages are used on a more regular basis and thus should have a larger base activation than the mental representations of category naming and color naming. In turn, more interference, and thus more control processes (cf. proportional control; Green, 1998; Meuter & Allport, 1999), should occur during language than task switching. Hence, differences in the size of behavioral switch costs do not necessarily indicate qualitative differences between language control and executive control. That is one of the main reasons why we included electrophysiological recordings; they allow us to make a more nuanced comparison between language and task switching with respect to their time course and neural underpinnings.

### Cue-Locked ERPs

In the early time window of the cue-locked ERPs, we observed a similar pattern across language and task switching paradigms: a posterior switch positivity in parallel with an anterior switch negativity. This pattern could partly be explained in terms of the early, posterior switch positivity that has been observed in task switching studies (Eppinger et al., 2007; Manzi et al., 2011); however, this positivity is generally not accompanied by an anterior switch negativity. A more straightforward explanation of the interaction between Trial type and Anterior/Posterior in the early time window is that it reflects the start of the same dipolar pattern we observed in the following time window (350–700 ms). This explanation seems to be confirmed by the ERP waveforms (see Figure 4), where the same ERP pattern continues from the early time window into the late time window. So, it might be that the early and later cue-locked time windows reflect the same protracted underlying process.

This long-lasting dipolar pattern, with a larger negativity over anterior sites and a larger positivity over posterior sites in switch than repetition trials, is reminiscent of the pattern observed in the task switching study of Lavric et al. (2008). This pattern could also explain the seemingly diverging, but temporally overlapping, patterns in the language switching studies by Lavric et al. (2019), who observed a posterior switch positivity, and Verhoef et al. (2010), who observed an anterior switch negativity, as two parts of the same dipolar ERP pattern. Since the posterior switch positivity and the anterior switch negativity observed in our study probably rely on the same underlying process, it is not exactly clear whether the dipolar pattern should be explained in terms of task rule activation (indexed by the posterior switch positivity) and/or goal maintenance (indexed by the anterior switch negativity).

The fact that we observed the same dipolar pattern in our language switching data as Lavric et al. (2008) observed in their task switching data is an indication that our pattern might represent a domain-general process. This hypothesis was further supported by the fact that we observed the same patterns across methodologically similar language and task switching paradigms. The Bayesian Null Hypothesis Testing provided further evidence for a similar dipolar pattern between paradigms with the exception of Fz in the later time window, which proved inconclusive. While this inconclusive finding leaves open the possibility that language control might not be entirely domain general, not too much weight should be put on this finding, as we did not find a significant switch cost difference between language and task switching in the cue-locked ERPs.

### Picture-Locked ERPs

The interaction between Trial type and Laterality in the early time window of the picture-locked ERPs (200–350 ms) could be interpreted in terms of the N2 component, which has been observed in both language and task switching studies (e.g., Gaál & Czigler, 2015; Finke et al., 2012; Jackson et al., 2001; Kang et al., 2020). The N2 component is characterized by a short negative peak around 200–350 ms that is larger for switch than repetition trials, and most pronounced over anterior sites. However, the early switch-related negativity observed in our study does not appear to correspond to the typical N2 (see Figure 7). For example, there is no short peak in the picture-locked waveforms that resembles an N2 component, and the difference between switch and repetition trials seems to occur broadly across the scalp rather than being constrained to anterior sites. Not observing a typical switch-related N2 over anterior sites is not entirely surprising, since this component does not seem robust across studies (e.g., Christoffels et al., 2007; Martin et al., 2013; Peeters, 2020; Peeters & Dijkstra, 2018; Timmer et al., 2019; Verhoef et al., 2009). Instead, it looks like the pattern in the early time window of the picture-locked data may reflect the beginning of the pattern that we see in the late time window.

In the late time window of the picture-locked ERPs, we also observed a switch-related negativity, which is in line with the findings of Kang et al. (2020), Peeters (2020), and Peeters and Dijkstra (2018). According to Kang and colleagues, this pattern represents an N400-like component such that word meaning retrieval is more difficult during switch trials. This explanation makes sense applied to our study, since, similar to the N400 component, the pattern we observed was a large negativity with a broad scalp distribution. According to Peeters (2020; see also Peeters & Dijkstra, 2018), this late, switch-related negativity reflects a domain-general process, because the same pattern has been observed in task switching studies (for a review, see Karayanidis & Jamadar, 2014). Evidence along these lines is also substantiated in our study, since Bayesian Null Hypothesis Testing on the late time window of the picture-locked ERPs indicated that it was more probable that language- and task-switch costs were similar than that they were different.

Because most models assume that language and executive control overlap at the goal level, it is not entirely clear why we should have observed a similar switch cost pattern in the picture-locked ERPs during language and task switching. One possibility is that some goal level processing might continue after picture presentation, and/or control processes related to the goal level might spill over into processing levels that are implemented after goal processing. This would be in line with models that assume an overlap between language and executive control at the goal level (e.g., Green, 1998). While these claims might be true, it seems unlikely that these control processes would affect the late time window, where we still observed the same neural pattern for language and task switching. Another explanation might be that control processes occur at the motor level (i.e., activation of the speech articulators), which is also shared across domains. Several language and task switching studies have provided evidence for control processes at the motor level (e.g., Philipp et al., 2007; Philipp & Koch, 2016; Reverberi et al., 2015). However, this motor explanation cannot account for the earlier similarities across paradigms. Ultimately, a combination of the explanations given here might contribute to the overall similar language- and task-switch cost pattern observed in the picture-locked ERPs.

### Limitations

In order to closely match the setup for the language and task switching paradigms, we had to use linguistic tasks (i.e., naming a color or semantic category) in the task switching paradigm. This allowed us to directly compare the results obtained in the language and task switching paradigms, since the only difference was whether participants switched between languages or tasks, respectively. However, this design means that it is possible that control processes specific to lexical retrieval might also have been involved in the task switching paradigm. It should be noted that the possible language-specific control processes implemented during our task switching paradigm would be control processes implemented during single language processing, as participants were always using the same language in the task switching blocks. Previous studies investigating the control processes during single and mixed language processing have shown that they can be different (Abutalebi et al., 2008; Declerck et al., 2017; Declerck et al., 2020). So, comparing within- and between-language switch costs does not necessarily mean that the same control processes are used. Future research may indicate whether similar ERP patterns can be observed across language and task switching, in which the latter is completely void of any linguistic processes. The downside of such a study would be that the language- and task-switch costs would not be directly comparable due to differences other than the type of switching (e.g., different stimuli, responses, response modality, number of response alternatives, etc.).

Another possible limitation of the current study relates to power. We observed no significant differences between language- and task-switch costs in the ERPs. Yet, it is difficult to draw strong conclusions on the basis of null results. It might be that our study was underpowered to the point that we could not observe any switch-cost differences between language and task switching. We tried to address this issue by including Bayesian Null Hypothesis Testing, which allowed us to determine to what degree the Null hypothesis is preferred over the Alternative hypothesis (e.g., Aczel et al., 2018; Rouder et al., 2009; Wagenmakers et al., 2018). All but one of the Bayesian factors indicated that the Null hypothesis is at least three times more likely to explain our data compared to the Alternative hypothesis. So, we suggest that our study can provide meaningful insights into the claim of domain-general language control.

Finally, as with most studies that investigate bilinguals, it should be noted that the outcome of our study could be different for other types of bilinguals. As can be seen in Table 1, the current study relied on early bilinguals with a relatively high proficiency in both languages. Although our results could be different for late bilinguals and/or those with lower language proficiency, no research has provided evidence along these lines. Hence, it remains unclear whether language history and/or language proficiency has any impact on the connection between language control and executive control.

### Conclusions

In this first ERP study to directly compare language and task switching with everything else held constant, we observed evidence for domain-general language control. Such evidence was observed in both the cue-locked and picture-locked ERPs, since no significant differences were observed between the language- and task-switch costs. So, our study indicates that language control is, to some degree at least, part of the more general executive control process. These findings seem to best align with models that propose that language control relies (at least partly) on domain-general processes (e.g., Green, 1998; Roelofs, 2003; Thomas & Allport, 2000).

An overlap between language control and executive control has many ramifications. For instance, it allows for the idea that better language control through constant practice should also influence executive control (cf. bilingual advantage; e.g., Prior & MacWhinney, 2010; however, see, among others, Nichols et al., 2020). Beyond bilingualism, our findings also indicate that language production encompasses processes that are not specific to language (e.g., Nozari & Novick, 2017; Roelofs, 2003, 2021).

## ACKNOWLEDGMENTS

This project has received funding from the European Union’s Horizon 2020 research and innovation programme under the Marie Skłodowska-Curie grant agreement No. 840286.

## FUNDING INFORMATION

Mathieu Declerck, Horizon 2020 Framework Programme (http://dx.doi.org/10.13039/100010661), Award ID: 840286.

## AUTHOR CONTRIBUTIONS

**Mathieu Declerck**: Conceptualization: Lead; Data curation: Lead; Formal analysis: Equal; Funding acquisition: Lead; Investigation: Lead; Methodology: Lead; Project administration: Equal; Visualization: Supporting; Writing – original draft: Lead. **Gabriela Meade**: Conceptualization: Supporting; Formal analysis: Equal; Investigation: Supporting; Methodology: Supporting; Visualization: Lead; Writing – review & editing: Equal. **Katherine J. Midgley**: Conceptualization: Supporting; Project administration: Equal; Resources: Equal; Software: Equal; Supervision: Supporting; Visualization: Supporting; Writing – review & editing: Equal. **Phillip J. Holcomb**: Conceptualization: Supporting; Formal analysis: Supporting; Methodology: Supporting; Project administration: Equal; Resources: Equal; Software: Equal; Supervision: Equal; Writing – review & editing: Equal. **Ardi Roelofs**: Conceptualization: Supporting; Funding acquisition: Supporting; Investigation: Supporting; Methodology: Supporting; Supervision: Supporting; Writing – review & editing: Equal. **Karen Emmorey**: Conceptualization: Supporting; Funding acquisition: Supporting; Investigation: Supporting; Methodology: Supporting; Project administration: Supporting; Resources: Supporting; Supervision: Lead; Writing – review & editing: Equal.

## TECHNICAL TERMS

Switch costs: The difference between switch and repetition trials.
Posterior switch positivity: Switch trials elicit a larger positivity across posterior electrodes than repetition trials.
Anterior switch negativity: Switch trials elicit a larger negativity across anterior electrodes than repetition trials.
