# Supplementary material for: On the Connection Between Language Control and Executive Control—An ERP Study
Source: Neurobiol Lang (Camb). 2021 Dec 23;2(4):628–46. doi: 10.1162/nol_a_00032 (PMC10158610; doi:10.1162/nol_a_00032)
Supplement: Supplementary file 1 [file nol-2-4-628-s001.docx]

Although we found no overall switch cost differences between language and task switching in the analysis of mean reaction times, it might be that there were differences among different components of the reaction time distributions. To examine whether this was the case, we performed ex-Gaussian analyses on the reaction time distributions. An ex-Gaussian analysis decomposes the reaction time distributions into two distinct components, namely a normal component with parameters mu (μ; mean of the normal distribution) and sigma (σ; standard deviation of the normal distribution), and an exponential component with parameter tau (τ; mean and standard deviation of the exponential, characterizing the tail of the distribution). Similar to Calabria et al. (2015), the μ and τ components were calculated for each of the four conditions per participant. We calculated the μ and τ components using on the retimes package (Massidda, 2013) in R. These μ and τ values were then used in separate analyses with the same independent variables as in the main reaction time analysis (i.e., Paradigm [language vs. task switching] and Trial type [switch vs. repetition trials]).

In the statistical analysis of μ values, a significant main effect of Trial type was found, with larger μ values in switch trials (823 ms) than in repetition trials (780 ms), *F*(1, 23) = 12.58, *p* = .002, ηp² = .354. No switch cost differences were observed between language and task switching (95% CI = [-38, 41]). Using Bayesian Null Hypothesis Testing, we confirmed that a model that does not include the interaction between Paradigm and Trial type, but does include both main effects, accounts for the data better than a model that includes the interaction (BF_01_= 3.55).

In the analysis of τ, no significant effects were observed (*p*s > .738), and thus no switch cost differences were observed between language and task switching (95% CI = [-30, 30]). Using Bayesian Null Hypothesis Testing, we confirmed that a model that does not include the interaction between Paradigm and Trial type, but does include both main effects, accounts for the data better than a model that includes the interaction (BF_01_= 3.87).

Taken together, these results provide additional evidence that switch costs were similar across the language and task switching paradigms.

References

Massidda, D. (2013). Retimes: Reaction time analysis. R package. https://rdrr.io/cran/retimes/man/ex-gaussian.html
